# Hypertension treatment in the oldest-old: focus group interviews with Swedish general practitioners

**DOI:** 10.1080/02813432.2022.2139436

**Published:** 2022-11-15

**Authors:** Marjo Berkhout, Kristina Bengtsson Boström, Anna-Lena Östberg

**Affiliations:** a Department of Public Health and Community Medicine/Primary Health Care, Institute of Medicine, Sahlgrenska Academy, University of Gothenburg, Gothenburg, Sweden; b Närhälsan Norrmalm Health Care Centre, Skövde, Sweden; c Research, Development, Education and Innovation Centre, Primary Health Care, Region Västra Götaland, Skövde, Sweden; d Department of Behavioral and Community Dentistry, Institute of Odontology, Sahlgrenska Academy, University of Gothenburg, Gothenburg, Sweden

**Keywords:** Focus group interviews, general practitioners, hypertension, oldest-old, physicians, primary health care, qualitative research

## Abstract

**Objective:**

This study explored the considerations and experiences of Swedish General Practitioners (GPs) of hypertension treatment in patients 80 years and above.

**Design:**

Qualitative design with focus group interviews. Data were analysed by qualitative content analysis.

**Setting:**

Primary health care centres (PHCCs), both rural and urban, in the Region of Västra Götaland, Sweden.

**Subjects:**

GPs and GP trainees working at PHCCs in 2019 and 2020. Five focus group interviews with 24 physicians were performed.

**Main outcome measures:**

Considerations and experiences of hypertension treatment in the oldest-old.

**Results:**

Eighteen GPs and six GP trainees participated in the study. The latent content was formulated in a theme: ‘The physician’s decision-making in the treatment of hypertension in the oldest-old implies the inclusion of both medical and humanistic considerations.’ The manifest content constituted three main categories: ‘The patient characteristics’ included medical condition, behavioural factors and daily life. ‘The physician’s role’ described the GP as a professional and her/his experienced support. ‘The treatment decision’ considered these categories and involved risk-benefit balancing and communication. For the future, the participants proposed better guidelines for the oldest-old multimorbid patients, increased teamwork, continuous cooperation with nurses and better cooperation with hospital physicians.

**Conclusion:**

Hypertension care for the oldest-old was experienced as complicated by GPs, due to the need of balancing medical and humanistic considerations. The GP’s clinical experience and the received support were of importance when making the treatment decision based on risk-benefit balancing and communication with the patient.

**Key points**
- GPs experienced the task of caring for the oldest-old patients with hypertension as complicated.
- Patient factors like multimorbidity, polypharmacy, behavioural factors and the patient’s condition of daily life were identified.
- Clinical experience and the experienced support at the PHCC were discussed as important for the GPs’ treatment decision.
- Treatment decisions for the oldest-old patients with hypertension were based on risk-benefit balancing and communication with the patients.

## Introduction

Life expectancy is increasing in Western populations. Consequently, the prevalence of conditions, such as hypertension, increases as higher age is associated with this condition [1]. Maintained health and quality of life with increasing age have paralleled the increasing life expectancy [2]. Older age is nowadays not inevitably associated with multimorbidity or frailty, increasing the complexity of treatment for many conditions including hypertension. Treatment recommendations for elderly with hypertension have been revised as studies showed a benefit of treatment of high systolic blood pressure (BP) [3]; however, the pharmacological treatment options were the same as for younger patients [4]. Frail and elderly patients are recommended individualized treatment [5].

In Sweden, as in other countries, most oldest-old patients (≥80 years) with hypertension are treated by general practitioners (GPs) [6]. In a recent study in Swedish primary health care (PHC) and hospitals, 80% of patients ≥80 years suffered from multimorbidity [7]. As most clinical trials exclude patients with multimorbidity, there is a lack of evidence regarding treatment and BP targets in these patients [8]. This lack of recommendations is problematic and GPs are challenged when making treatment decisions [6].

Dutch GPs were reluctant to start hypertensive treatment in patients ≥80 years; however, cardiovascular disease and diabetes were reasons for drug treatment [9]. This illustrates the dilemma of the physicians; both to reach BP targets and avoid cardiovascular complications [10], and to avoid overtreatment and side effects [11].

Surveillance of Swedish health care performance is done using quality registers with data from medical records, for instance, BP values and cardiovascular complications. Register data [12] are available to staff in PHC and comparisons can be made between different PHCCs. At many PHCCs, registered nurses work with physicians in teams performing BP check-ups and monitoring life-style changes [13].

Several guidelines for hypertension treatment exist: international, national, regional and local. Many GPs are uncertain about which guidelines to use and experience them as insufficient for the treatment of old multimorbid patients [6,14,15]. Studies of withdrawal of drug treatment for hypertension in the oldest-old could not show whether this is beneficial or not, due to insufficent quality of the studies [16]. This leaves the individual GP to decide in cooperation with the patient whether treatment should be started, intensified or terminated. We aimed to explore the current considerations and experiences of Swedish GPs of hypertension treatment in patients ≥80 years.

## Methods

### Design and setting

A qualitative design with focus group interviews was used and data collection was performed 2019–2020 at PHCCs in Region Västra Götaland, Sweden.

### Participants

GPs and GP trainees, with at least one year of PHC experience, working in public or private PHCCs were strategically selected in urban and rural areas. Locum physicians and interns were excluded. Participants from the same PHCC constituted a focus group.

### Ethics

The Swedish Ethical Review Authority (Reg. no.: 2019-03350) had no objections to the study. All participants gave written consent to participate.

### Data collection

Focus group interviews were performed at the participants’ own PHCC and took place during scheduled meeting times. One of the authors (MB), researcher and clinically active GP, moderated all the groups. The third author (ALÖ), researcher with experience of qualitative methods and a dentist, observed verbal and non-verbal communication and took notes.

An interview guide was developed [17] based on prior studies and clinical experience of the two first authors. The main topics to be discussed were: (i) physicians’ reflections on hypertension treatment in patients ≥80 years, (ii) factors that physicians perceived influenced their decision-making when starting, intensifying or discontinuing hypertension treatment and (iii) physicians’ thoughts on how they could be supported in the treatment of the elderly with hypertension. All interviews were opened by the same question: What do you think about hypertension treatment in the oldest patients? The participants could then discuss freely and the moderator used probing questions, when needed.

At the end of the interview, the participants had the opportunity to comment on the topics or introduce new ones. Finally, the observer asked questions based on the observations. When the participants had left, the observer and the moderator reflected on whether new topics had arised. Additional focus group interviews were performed until the main topics were deemed sufficiently illuminated and no new topics were introduced [18].

The duration of the focus group interviews varied between 43 and 53 min, were sound-recorded and transcribed verbatim by the moderator. Statements of the participants illustrate the findings and are indicated in the text with the focus group in which they participated and assigned an individual number (e.g. FG 3:2).

### Data analysis

In addition to the two authors involved in the data collection as described above, the second author (KBB), who is researcher and GP with experience in the treatment of hypertension and a member of the Regional Medicines Committee, participated in the analysis. The authors analysed the data separately and then met repeatedly to discuss the interpretations and to reach consensus.

Qualitative content analysis was used [19], searching for both overt (manifest) content and non-spoken underlying (latent) content. The visible/obvious components of the participants’ outspoken statements constitute the manifest content. The latent content was revealed by a deeper interpretation of the underlying meaning of the statements [19].

First, meaning-bearing units were identified, i.e. words or statements with common signification. These units were condensed; that is, shortened by preserving the core content. The condensed meaning-bearing units were further shortened and assigned codes. The codes with similarity of content were grouped into subcategories and labelled close to the content of the original transcribed data. In the next step, the subcategories were abstracted to a higher level, to categories. In the final step of the interpretative analysis, an overall theme was sought to represent the underlying latent content i.e. a deeper understanding of the data [20].

## Results

Five focus group interviews with four-six participants were performed including 24 physicians, 18 GPs and 6 GP trainees. Fourteen of them had received their medical education in Sweden and the majority of the others in the EU. Their age ranged from 29 to 72 years, 13 were women. The clinical PHC experience ranged from one and a half to 36 years. Two interviews were performed at rural and three at urban PHCCs, one of them privately run. During the interviews, most of the topics in the interview guide were raised spontaneously.

**Figure 1.**
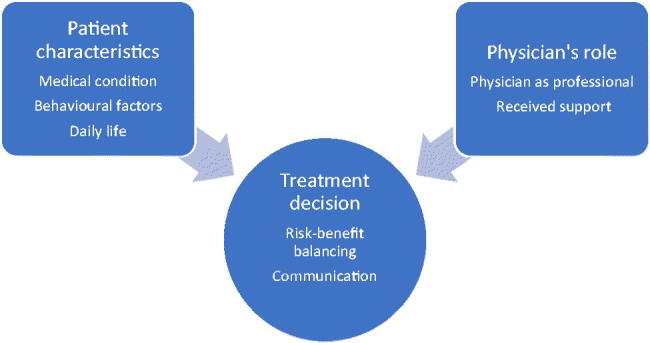
Main categories and subcategories.

Three main categories together with subcategories, comprising both manifest and latent content on varying levels of abstraction are presented below. Two of the main categories ‘Patient characteristics’ and ‘The physician’s role’ formed the basis for the third category, ‘The treatment decision’ (Figure 1). A theme with latent data was formulated as: ‘Physicians’ decision-making in treatment of hypertension in the oldest-old implies the inclusion of both medical and humanistic considerations.’ The medical considerations could be described as a view on the individual parts at organ and body function level, influencing the medical decision-making based on former disease, risk of complications, clinical measures and laboratory results. The humanistic considerations include a holistic view, meaning that the patient is seen as a whole person where the physician takes the patient’s life situation and quality of life into account while making therapeutical considerations, including the risk of adverse-events, side-effects and interactions.

### The patient characteristics

This main category mostly contained manifest content of the interviews. Three subcategories were identified: ‘Patient’s medical condition’, ‘Patient’s behavioural factors’ and ‘Patient’s daily life’.

#### Patient’s medical condition

The patient’s medical condition included the presence or absence of physical and/or mental diseases. It was commented that many of the oldest-old patients not only suffer from hypertension, but also have other chronic physical conditions, for instance, impaired kidney function, heart failure, and a great number of other diseases and polypharmacy with risk for interactions with antihypertensives:

*They also suffer from multimorbidity, which is a problem; many other diseases, perhaps a prior stroke, which complicates matters as it’s not just hypertension* (Focus group (FG) 4:4).

A specific example that was mentioned was if an elderly man already had medication for benign prostate hyperplasia (α1-receptor blocker), then this could affect the antihypertensive treatment. Another aspect, frequently brought up, was when an elderly patient suffers from dizziness and needs antihypertensives, then the medication increased the risk of falling, possibly leading to fractures:

*…but when a male patient is already undergoing prostate treatment with alfa-blockers, then you have to consider the blood pressure-lowering effect of those and the risk of hypotension and falls* (FG 2:3).

The participants described a wide variation in the oldest-olds’ mental condition, specifically how well their cognitive functions were preserved. There are patients with cognitive problems or dementia with gradual loss of autonomy who cannot always express themselves. However, there are also otherwise healthy hypertension patients who are really concerned about their BP and want the physician to give them the best possible care.

#### Patient’s behavioural factors

This category was further divided into two subcategories: ‘the patient’s lifestyle and selfcare’ and ‘patient compliance and adherence with recommendations and prescriptions.’

The participants identified risk factors in patients’ lifestyle, such as alcohol consumption, smoking and dietary habits, all contributing to the risk of cardiovascular complications. But they also noted that the habits of the oldest-old patients probably had prevailed during a long life and it might be a useless effort to try to change these habits, both for the patient and for the physician:

*I find elderly people smoking really difficult (FG 1:1) … Yes, if they still smoke when they are over 80, it’s probably a losing battle (FG 1:4).*

Alcohol consumption among elderly people was commented on in most of the interviews, for instance, old ladies drinking a lot of wine.

Regarding patient’s physical activity, participants experienced it as hard to give recommendations to patients with poor health. Patients in better health were more often given advice on physical activity. Those with poorer health receiving community care sometimes lacked access to support for physical exercise because of lack of staff. Patient adherence to lifestyle recommendations was generally experienced as poor:

*…but life-style, that’s a compliance problem (FG 3:5). Yes (FG 3:2) (….) cemented in a way that makes me feel powerless (FG 3:5).*

Likewise, the experience of several participants was that patients had poor adherence to prescribed medication and this was brought up in all interviews. They referred to their own experiences, especially observations made during home visits:

*…it often happens that I make a house call and find cupboards stuffed with medicines, unopened bottles that have been picked up at the pharmacy exactly every third month…(FG 4:4). Yes, that happens (FG 4:5).*

Still, the participants noted that starting antihypertensive medication could be a time-saving intervention compared with getting the patient to give up bad habits and following up lifestyle changes.

#### Patient’s daily life

The participants identified a wide variation of conditions of daily life within the group of the oldest-old, ranging from poor to very good. This issue was discussed in all the groups with varying intensity and included both the type of housing and the possibilities to live one’s life as desired. The participants often emphasized the importance of understanding the patient’s daily life and taking this into account. The impact of both diseases and medication on the patient’s daily life was easier to understand when the patient was receiving community care or living in a care home. This allowed frequent observations by the nurses and assistant nurses who could then provide the physician with information. Also, family or other persons in the patient’s social network could help the physician better understand the patient’s condition of daily life. The participants found it more difficult to gain this understanding of the oldest-old without a close network of medical staff, family or people with whom they have regular contacts:

*…in home nursing and care homes, the assistant nurses see the patients every day, possibly make two or three house calls every day. …. Their reports become more objective than the patients’ own, when they live alone and can’t really tell whether they are dizzier today than yesterday when standing up …. so the reporting is totally different (FG 1:2).*

The possibilities of having a good quality of life with a ‘gilt edge’ must be considered, according to the discussions:

*‘’…they must have a kind of gilt edge too.’’ (FG1:4)*

*Maybe they could live as they like during their last few years, have a good life and be happy and eat cake if they want to (FG 3:4).*

### The physician’s role

This category describes the physician’s role in taking care of the oldest-old with hypertension and was divided into two subcategories: ‘The GP as a professional’ and ‘Received support’. The participants considered the assignment of taking care of the oldest-old with hypertension to be rather complicated. Professional experience was pointed out as important for the task in all focus groups.

#### The GP as a professional

Some of the participants, with long clinical experience of PHC, especially those who had been working for a long time in Sweden, reported that they primarily relied on their own clinical experience when taking care of the oldest-old with hypertension:

*…I think our medical knowledge is put to the test in some way. We are trained to manage the oldest-old and their hypertension (FG 4:1). Exactly (FG 4:3). … it’s a challenge, but a rather exciting one. It still feels like a more interesting part of the job (FG 4:1).*

They noted that recommended target BP levels have changed over time. Moreover, they pointed out that the guidelines do not take all the circumstances of the individual into account, and this is a challenge for the physician. The considerable interest in the topic of hypertension could be deduced, in part, from the discussions, especially as some physicians had taken special responsibility for hypertension care at their PHCC.

Time, or rather, the lack of time, for the professional role was commented on. Telephone calls must be made, laboratory tests ordered and documentation kept in the patient’s medical record and so on. The time for follow-up and reflection is limited.

#### Received support

The support given to GPs in the task of taking care of the oldest-old with hypertension was divided into two subcategories: ‘Formal support’ and ‘Informal support’. Table 1 shows an example of the qualitative content analysis resulting in the category ‘Received support’.

**Table 1.** Example of qualitative content analysis resulting in the category 'Received support’.

Condensed meaning units	Codes	Sub-categories	Category
Teamwork scheduled around patient (*nurse - assistant-nurse -physician*)	Organizational conditions	Formal support	Received support
Access to guidelines			
Access to technical equipment *(i.e. home and/or 24-h blood pressure devices)*	Resources		
Access to continuing education			
Consulting and discussing with colleagues at the PHCC	Experienced colleagues at the PHCC	Informal support	
Receiving information from and consulting with nurses in community care	Cooperation with staff at other care- giver establishments		

Some PHCCs in Sweden have access to registered nurses working in hypertension out-patient clinics. These organisational conditions involving teamwork were discussed by the participants as contributing to the experienced formal support in four of the focus groups. The conditions for this support are controlled by the local management and may vary between PHCCs:

*If it is just hypertension, they see the registered nurse every second year and the physician the intervening years…. so, it’s very good cooperation (FG 5:2).*

A desire was expressed for additional resources for out-patient hypertension clinics in cooperation with registered nurses who could be delegated some tasks by the physicians.

Formal guidelines for hypertension treatment were discussed and most physicians regarded these as insufficient for the oldest-old multimorbid patients. However, the guidelines provided general advice and less experienced physicians sought more often support in the guidelines. But the issue is complex when it comes to the individual patient:

*…sometimes it’s hard to marry the guidelines with reality when you have a patient in front of you who also suffers from other conditions (FG 2:3).*

The participants had rarely studied the scientific basis for the guidelines and the main reason stated was lack of time, which was reported in all focus groups. Furthermore, as there are guidelines at different levels – international, national, regional and local – it was stated as hard to know which guidelines to follow. Many physicians mentioned that they would appreciate consensus on the use of national guidelines.

There were some differences in resources between the participating PHCCs. One example was access to formal technical support, for instance, ambulatory BP monitoring (ABPM). The participants expressed their wish for access to ABPM, but the PHCC lacked the technical ability to document the result in the medical records due to software incompatibility. This could lead to some frustration:

*The technology prevents that, or rather our medical record system, I think it’s very unfortunate (FG 4:3). Okay, so I said, ‘leave it’, because I can’t be bothered …. (FG 4:4).*

In other PHCCs, the participants were satisfied with having access to home BP devices with automated recording to lend to patients for measuring BP at home over a period of time. Technical solutions for direct data transfer from home BP devices or ABPM to the patient’s medical record were discussed to be an improvement.

Resources for continuing education also varied between PHCCs according to the discussions.

The subcategory ‘Informal support’ included ‘Experienced colleagues at the PHCC’ and ‘Cooperation with staff at other caregiver establishments.’ In two of the PHCCs, one physician was designated to specialise in hypertension and be accessible for consultation, to discuss patient-related questions and take responsibility for educating other staff members working with hypertension care. Experienced colleagues at the PHCC were important for informal consultation.

Increased teamwork and separate geriatric care out-patient clinics for the oldest-old patients were suggested as future improvements of hypertension care.

### The treatment decision

When deciding on treatment for the oldest-old patient with hypertension, the patient-related and physician-related factors described here played an important role. Two subcategories were identified: ‘risk-benefit balancing’ and ‘communication’, often taking place as parallel processes.

#### Risk-benefit balancing

Balancing the risks and the benefits of hypertension treatment for the individual oldest-old patient was regarded as one of the most challenging tasks by the participants. In most cases, several different factors had to be considered. The patient’s medical condition and other medication were naturally the starting point that had to be balanced against the risk of potential side effects and interactions:

*If it’s only hypertension (laughs) it is very easy, but if they suffer from multimorbidity, it is a major challenge. Yes, they need combined treatment, experience side effects, there are some drugs that raise the blood pressure, while others lower it. Some affect kidney function… (FG 2:2).*

When severe hypertension was present, it was considered particularly important to start or modify the antihypertensive drug treatment. The estimated benefit was then seen as clearly outweighing the potential risks of side effects. If the patients had additional conditions, such as diabetes or a previous stroke, physicians were even more eager to start or intensify medication.

The reasons mentioned for reducing or discontinuing hypertensive medication were when an oldest-old patient received palliative care, was mainly lying in bed or was suffering from dizziness and in risk of falling. Antihypertensives were then considered to be of little benefit:

*If a patient has a great risk of falling, this could be a reason to refrain from treatment or at least discontinue some antihypertensives (FG 2:4).*

There were no differences in treatment goals for hypertension in the oldest-old based on the patient’s living situation; that is, whether receiving home care, living in a nursing home or living independently at home.

Many participants expressed a more holistic perspective considering side effects as risk of orthostatic BP or impaired kidney function leading to slower degradation of pharmacological substances.

There was also another aspect to risk balancing; that is, the physicians were aware that the achievement of BP targets in their own PHCC might be compared with other PHCCs and regional targets. In one focus group, the participants mentioned that they were aware of their own performance regarding the statistical outcome for BP targets in their own PHCC and did not want to perform worse than a neighbouring PHCC.

#### Communication

Communication took place between different actors; between the physician and the patient; between the physician, the patient and family/close contacts and between the physician and other caregivers.

Most physicians were sensitive to the patients’ expectations and views on hypertension treatment, but when the severity of hypertension with consequent risk of complications increased, less attention was paid to patient’s preferences to treat or not to treat. Furthermore, the patient needed to be informed of the risk of no treatment and how to monitor imminent side effects:

*Well, it depends, if they have a blood pressure of 210/115 (mmHg), we have to prescribe medication whether the patients want it or not. You have to inform them about the risks of not treating … (FG 3:4). Actually, I don’t think I give them a choice. If the blood pressure is that high, there’s not much to discuss (FG 3:2).*

However, participants said that they may refrain from medication according to the patient’s preferences:

*…or if the patient refuses, despite information about the risks and says: ‘No I don’t want that crap’. Then there’s nothing to do, of course, …. You have to let it go (FG1:2). But it happens very rarely (FG 1:4).*

The patients’ mental state was recognized as important for communication, specifically how well their cognitive functions were preserved and thus their ability to participate actively in the discussion about their antihypertensive treatment. Some patients might urge the physician to prescribe antihypertensive treatment. In such cases, the participants emphasized the importance to take the patient seriously and discuss optimal treatment. They also noted that with today’s increasing life expectancy, patients can retain good health into old age, but when they fall ill, many have high demands regarding their care:

*It may be a 95-year-old who is very worried when the blood pressure is slightly elevated, because he/she knows it should be below 140 (mmHg), and then you have to do more (FG 4:2)… but also relatives who are worried, because they want to have their parents around until they die themselves, so they put pressure on you (FG 4:4).*

On the other hand, there are frail patients who have difficulties expressing themselves. If the physicians are unable to elicit the patient’s own preference, they have to rely on information given by family or staff members in nursing homes who may have contradictory views on pharmaceutical treatment. There is a risk that the parties involved, when making decisions for the oldest-old, fail to take this into proper account. This poses a complicated ethical problem that generated engaged discussions especially in one of the focus groups:

*But I believe that autonomy, is the most important of the ethical principles in health care… (FG 5:1). Yes, but my spontaneous reflection is that less attention is paid to autonomy, it’s not considered as much when people are older… (FG 5:4).*

Generally, communication with staff or family concerning the patient was not experienced as problematic.

The transfer of information and communication between GPs at the PHCC and hospital physicians concerning the patient’s condition and medication was experienced as insufficient. For example, patients seeking hospital care returned to the PHCC with new drugs. In many cases, the physicians at the hospital had not considered the patient’s situation as a whole and the GP had to modify the treatment:

*when he comes back to the PHCC, I stop it. No, I don’t do that, but I lower the dose, I adjust it, so he gets a tolerable existence (…). Sometimes it feels like there’s no communication at all between hospitals and primary care (FG 5:1).*

The fact that medical records are kept separately and are not communicated between caregivers (PHC and hospital care) was seen as a major disadvantage. One common medication list that is available to all caregivers was suggested by the participants as a possibility to improve hypertension care and reduce the risk of mistakes, double medication (generics and branded drugs) and reintroduction of former medication:

*Information transfer is always a problem. I don’t know how to improve that but, as we’ve said before, patients are admitted to the hospital and are given drugs they may have been tried before. Then they’re discontinued and a year later, the patient is admitted again and put on the same medication again (FG 4:5).*

## Discussion

### Principal findings

The main finding was that hypertension care for the oldest-old patients was experienced as challenging for physicians. The physician must deal with the often complex balancing of medical and humanistic considerations as intertwined concepts and not in opposition, when making treatment decisions.

### Strengths and limitations

Focus groups interviews were chosen for data collection, having the advantage of interaction between the participants and reflection on different considerations and experiences, compared with individual interviews [21]. Both rural and urban settings were represented in the sample as well as the mode of operation (public and private). The participants were recruited in four geographic areas within the Västra Götaland region to represent both rural and urban settings [21]. An advantage with participants from the same PHCC, that is pre-existing groups, was that they shared their daily life and were used to discuss topics together [22]. We chose to include both GPs and trainees from the same PHCC, despite that the trainees may have been more reluctant to express their views in front of more experienced colleagues/supervisors [23]. A risk was if conflicts or hierarchal structures existed within the group, this could affect the interviews negatively. The moderator was aware of the risks and if a participant had been quiet for a time, she/he was encouraged to express views on the topic discussed. Since participation was voluntary, physicians who did not want to express their opinion, probably refrained from participating and ethical aspects prevented more questioning in the matter.

The number of groups was decided to suffice after five interviews. The basis for this decision was that the findings were gradually confirmed throughout the data collection process until adequate data for the analysis was deemed to have been gathered and no new topics emerged in the discussions [24].

In the chosen method for analysis, qualitative content analysis, the trustworthiness of the results is evaluated regarding credibility, dependability and transferability [19]. The credibility criterion; that is, how well the collected data and analysis process match the focus of the study, was fulfilled by including focus group participants of various background, age and experience and by interview questions stimulating interaction between the participants [23]. Similarities and differences between categories were discussed repeatedly between the researchers [19,25], which strengthens the credibility of the study. The three researchers’ different experiences and perspectives also contributed to the depth of the analysis, whereas the level of abstraction and the depth in the analysis varied in both manifest and latent interpretations.

To meet the criterion of dependability; that is, dependent of external circumstances as the extent to which the data collection and the researchers’ decisions during the analysis are consistent over time, the study was carried out during a limited period of time. An interview guide was followed and no new guidelines on hypertension were presented during the data collection period. Thus, the criterion was considered to be met [20].

The transferability criterion can be met by giving a description of the context, the characteristics and the selection of participants, the collection of data and the analysis process [19]. In our study, we recruited PHCCs from different areas in the region, which were presumed to have different conditions, to capture the width of both common and alternative experiences and considerations of the participants. The findings can be transferred to similar contexts in Swedish health care but probably also to other countries with similar hypertension care for the oldest-old.

### Findings in relation to other studies

The patient characteristics and the treatment decision were intensively discussed in a down-to-earth way in the focus groups. The participants’ own role as physicians was partly commented on directly, but their reflections on this subject were less explicit; i.e. constituted latent content. Since physicians often discuss patient issues, it was probably easy to do that in the focus groups as well, but it was more unusual for them to reflect on their own role when making decisions.

An asymmetrical balance of power could be seen between the patient and the physician, as also described in the Norwegian focus group study among GPs [26]. The physician in his/her professional role always has the choice to decide on antihypertensive treatment, while the patient has a secondary choice and is dependent on the physician’s ability for shared decision-making and treatment proposals. This was particularly clear when the physicians in the focus groups discussed treatment options for patients with severe hypertension; here they showed a clear preference for a paternalistic approach. In an Australian study, some GPs perceived that older patients wanted the doctor to make treatment decisions for them i.e. expected a paternalistic relationship [11]. Both GP strategies can lead to an asymmetrical balance of power between the patient and the GP.

Another asymmetrical balance of power could be identified, between physicians working at the hospital and those in PHC. According to the participants, hospital physicians rarely communicated with GPs about the patient’s medication and tended often to change or reinstate the patient’s medication. They did not always evaluate the potential side effects and consequences for the patient’s daily life as also described in an English study [27].

The possibility to transfer knowledge and empower the patient, i.e. to support lifestyle changes, was earlier identified as an advantage of working in a team [28], as this approach can improve quality of care [13]. This was mentioned in four out of the five groups. In concordance with the Australian study mentioned above, the participants gave recommendations on lifestyle changes more often to healthier older patients [11]. Burdening the frail patient with strenuous lifestyle recommendations was though not mentioned overtly as an argument in this study as it was in previous studies [29].

According to the participants, a high chronological age was no reason to refrain from starting treatment with anti-hypertensives, in contrast to findings in previous studies [9,30].

### Meaning of the study

Hypertension care for the oldest-old was experienced as complicated by GPs, due to the need to incorporate a balance between medical and humanistic considerations. The GP’s clinical experience and received support were of importance, when deciding on treatment on the basis of risk-benefit balancing and communication with the patient. The interviewed physicians wished to improve hypertension care for the oldest-old and wanted more specific and holistic guidelines, taking multimorbidity, polypharmacy and conditions of the patient’s life into account. Increased teamwork with registered nurses and shared medication lists with the hospitals, to avoid unnecessary reinstatement of former medication, were also mentioned as desirable to increase support for the GPs in their daily clinical practice when taking care of the oldest-old patients with hypertension.
